# Fabrication of composite ceramic polymeric membranes for agricultural wastewater treatment

**DOI:** 10.1038/s41598-025-85542-w

**Published:** 2025-01-17

**Authors:** Neamatalla M. Azzam, Sahar S. Ali, Gehad G. Mohamed, Mohamed M. Omar, Shereen K. Amin

**Affiliations:** 1 Chemistry Department, Faculty of Science, Cairo University, Giza, 12613 Egypt; 2 Chemical Engineering and Pilot Plant Department, Engineering & Renewable Energy Research Institute, National Research Centre (NRC), Giza, 12622 Egypt; 3 Nanoscience Department, Basic and Applied Sciences Institute, Egypt-Japan University of Science and Technology, Alexandria, 21934 Egypt

**Keywords:** Ceramic membranes, Heavy metals, Wastewater treatment, Polyamide 6, Composite membrane

## Abstract

Humans have contaminated water supplies with harmful compounds, including different heavy metals. Heavy metals can interfere with human and animal vital organs and metabolic processes. They are also persistent and bioaccumulative. So, this study aimed to fabricate composite ceramic membranes (CCM) from Egyptian raw substances to eliminate heavy metals from agricultural wastewater. A ceramic supporting (CS) filter constructed from ball clay, kaolin, feldspar, and quartz using corn starch flour as a pore-developing agent. CS fired at two different temperatures and soaking times. Then, a thin polyamide 6 (PA6) coating was dip-coated over the upper layer of the support membranes. The raw materials and prepared CCM were subjected to characterization and applied to treat agricultural wastewater from the Kitchener drain in Kafr El-Sheikh Governorate, Egypt. The results showed that the CCM (M2) (membrane sintered at 1000 °C/30 min soaking time and modified with PA6) had a higher pure water permeability of 558.5 L h^−1^ m^−2^ than the membrane (M4) (membrane sintered at 1100 °C/180 min soaking time and modified with PA6). The study examined how effectively the membranes removed toxic substances from wastewater. The findings exhibited an excellent removal of > 80% and up to 97.02%, > 80% and up to 99.97% of the heavy metals, and optimum fluxes of 341.07 and 276.35 L h^−1^ m^−2^ were achieved in the cases of M2 and M4, respectively. Furthermore, with a low flux decline ratio and a high permeate recovery of 92.3% for wastewater, the modified M4 membrane demonstrated remarkable antifouling capabilities.

## Introduction

One of the primary causes of environmental pollution is the discharge of industrial effluents into waterways. Because human waste is released into water bodies, wastewater is the primary cause of water pollution globally. This lowers the value of ecological water, rendering significant amounts of water unsafe for diverse exercises. The increase in the complexity of toxic discharge has coincided with the rapid industrial expansion of the preceding era. Therefore, treating all types of wastewater and polluted water is necessary because they contain many different pollutants, such as heavy metals. The importance of treating contaminated water is due to many reasons, including the scarcity of water resources and the widespread contamination of these sources with pollutants, which threatens humans and the environment^1^. Heavy metals (HMs) are non-degradable metals with high atomic weights and toxicity^2^. It also accumulates in living organisms, causes many health problems such as various types of cancer, kidney failure, and liver cirrhosis, and sometimes leads to death^3^. There are many sources of heavy elements that lead to the entry of these pollutants into the ecosystem, such as various industries, including the paint industry, mining, electroplating, battery manufacturing, electronics, dyeing, leather tanning, galvanizing, plastics, and pharmaceutical industry drainage, in addition to agricultural drainage, which contains pesticide residues and chemical fertilizer^4^.

Therefore, numerous advanced technologies are accessible for effectively treating heavy metal-contaminated water., such as water filtration technology using membranes^5^, ion exchange^6^, coagulation^7^, adsorption^8^, and electrochemical treatment^9^. The membrane treatment technique has many advantages, such as simple preparation methods, chemical resistance, high temperature, good mechanical properties, and high-efficiency separation^10^.

Ceramic membranes (CMs) are created using various costly raw materials, such as oxides (aluminum, titanium, zirconia, and silicon). Therefore, many researchers have used low-cost alternatives in addition to their abundance as natural raw materials, such as kaolin, feldspar, clay, dolomite, and quartz sand, in addition to using waste. For example, using rice husk and fly ash leads to the optimal utilization of resources as well as the conservation of the environment. CMs have been developed by adding thin layers of materials such as chitosan, polyamide, polyvinyl acetate, polysulfone, and many other materials, including nanomaterials. Polyamide has a low coefficient of friction, abrasion resistance, high tensile strength, and acceptable hardness. Several methods are used to apply these layers to the upper surface of the membrane, including grafting, spray and dip coating, and vapor deposition. One of the methods that has proven efficient in preparing ultrafiltration and microfiltration membranes is the dip coating method^11^.

The Kitchener drain is located in the central part of the Nile Delta and spans an area of around 1,800 square kilometers in total. The Kitchener drainage system originates at El-Gharbiya, located north of Tanta city. It flows towards the north through Kafr El-Sheikh to Baltim city, where it meets the Mediterranean Sea^12^. The Kitchener drain releases more than 1.9 billion cubic meters annually and flows from the south to the north, ending in the Mediterranean Sea. The water in the drainage system comprises 75% agrarian discharge water, 23% domestic water, and 2% industrialized water. It is contaminated by a variety of effluents that are released by homes and industrial applications. The aquatic environment is believed to be the primary factor influencing health and illness. According to El-Amier et al*.*^13^, several untreated raw water discharges are from the El-Gharbiya and Kafr El-Sheikh Governorates and other nearby cities and towns. The current work uses membrane separation technology to remediate Kitchener drain water^14^. This study addresses the primary issue of poor surface water quality in the Kitchener drainage system. Freshwater is a limited resource, so it is important to consider its scarcity. And its quality impacts numerous significant ecosystems, so evaluating water quality is crucial.

This research investigated the potential of fabricating low-cost ceramic-based membranes using local materials for application in agricultural wastewater treatment. In recent years, wastewater treatment has presented substantial challenges due to its inherent complexity and resistance to traditional treatment procedures. To improve the membranes’ physical, mechanical, and filtration properties, two groups of ceramic membranes were prepared and sintered at two different temperatures: 1000 °C and 1100 °C. Then, they are coated by thin layer of polyamide 6 and ethylene diamine to improve their wastewater treatment capabilities. This work highlights the possibility of constructing innovative, efficient and economically viable membranes for agricultural wastewater treatment utilizing low-cost materials. The mechanical strength, water absorption, pore size, flux, and separation efficiency of heavy metals from the drainage effluent are investigated to assess the efficiency of the CCMs.

## Materials and procedures

### Materials

Ball clay and grog (fired clay) were provided from the Quarries of Upper Egypt (Aswan, Egypt). Potash feldspar (KAlSi_3_O_8_) and quartz sand were collected from the Eastern Desert (Egypt). Kaolin was obtained from the South Sinai (Egypt). Starch was purchased from local markets in Egypt. Extra pure polyvinyl alcohol (PVA) (C_2_H_4_O)n, used as a binder, was purchased from Oxford Company. Polyamide 6 (PA6) (C_6_H_11_NO)n, (Nylon 6) was purchased from Dop Organik Kimya. Ethylene diamine (EDA), C_2_H_8_N_2_, was purchased from Sigma-Aldrich. Formic acid (FA) CH_2_O_2_, with 85% purity, is used as a solvent to dissolve PA6 and is manufactured by El Nasr Pharmaceutical Chemicals Company. No additional purification was required because all the chemicals were analytical grade and used as supplied. All experimental procedures were carried out using de-ionized (DI) water.

### Methods

#### Preparation of the raw mix

A mixture of clays “ball clay and kaolin” (46 wt.%), potash feldspar (KAlSi_3_O_8_) (20 wt.%), quartz sand (9 wt.%), and grog (fired clay) (25 wt.%) was prepared. Starting materials were ground into fine particles in a laboratory ball mill for half an hour.

#### Preparation of PVA and PA6 solutions

A precisely measured amount of PVA material was slowly added to heated water on a hot plate with a magnetic stirrer to create a 3% PVA solution by weight. The liquid was mixed constantly until the PVA was completely dissolved, after which the solution was allowed to cool for one hour.

A specific amount of PA6 material dissolved precisely to create a 20% solution by weight, slowly added to 79% by weight formic acid and 1% ethylenediamine (as an additive) in an ice bath while mixing continuously. Continuously mix the solution until the PA is completely dissolved for optimal results, and then the solution is left in a beaker inside an ice bath until clear. Therefore, it is ready to use.

#### Fabrication of CS membranes

Cylindrical disk samples with a diameter of (50 ± 2) mm and a thickness of approximately 5 mm were formed by pressing 20 g of the mixtures (fine raw mix powder with 5% by weight starch and 20% by weight PVA solution (3% concentration) that was previously prepared) in a stainless-steel mold using a hydraulic compress under a uniaxial load of 30 MPa; the CSs were dried in two stages using a drying oven. The initial step was for six hours at 60 ºC, followed by incubation for another six hours at 110 ºC. The CSs were sintered using a muffle furnace under two distinct conditions. The membrane specimens were sintered at 1000 ºC and soaked for 30 min (M1 and M2). The membrane specimens were fired at 1100 ºC with a 180-min soaking time (M3 and M4). The heating rate was maintained at a constant 5 ºC min^−1^ to ensure precise and reliable results. Similarly, cubic specimens with dimensions of approximately (50 × 50 × 50) mm^3^ were molded, dried, and fired under the same conditions as those used for membrane specimen preparation to assess the fabricated CM’s compression strength.

#### Modification of ceramic membrane surfaces

**Fig. 1:**
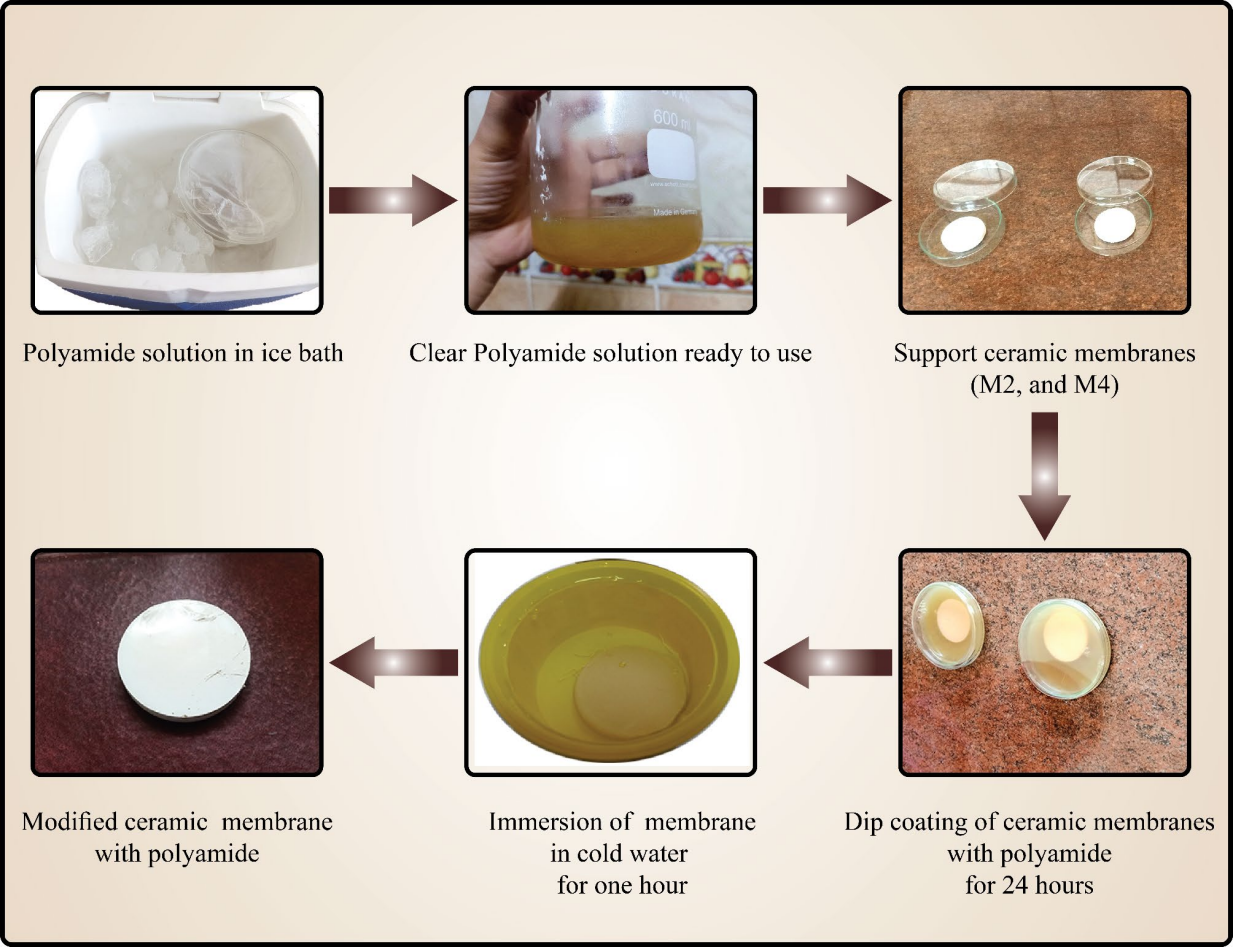
Ceramic membrane modifications.

Membranes sintered at different temperatures (M2 and M4) were modified using the dip coating. They were submerged in a PA6 solution of 20 wt.% for 24 h. Then, after removal from the PA6 solution, they were immersed directly in a cold-water bath for one hour. Finally, the samples were rinsed with distilled water multiple times to ensure the highest standard of cleanliness. The modification procedures are shown in Fig. 1.

#### Characterization

##### An analysis of the characteristics of the raw materials

The raw powder’s chemical composition was analyzed using a PANalytical AXIOS wavelength dispersive (WD-XRF) spectrometer installed at NRC, with X-ray fluorescence spectroscopy (XRF). X-ray diffraction (XRD) analysis was performed using a BRUKUR D8 advanced computerized X-ray diffractometer installed at the Central Metallurgical Research and Development Institute (CMRDI) to determine the mineralogical composition of the raw materials. Thermogravimetric analysis (TGA) and differential scanning calorimetry (DSC) examined the starting material powder’s thermal behavior. The studies were performed in an atmosphere of nitrogen at an ambient temperature of 25 °C to 1000 °C, and the heating rate was maintained at 10 °C min^−1^ using a Setaram THEMYS ONE+, installed at NRC. The manufacturer’s software processed the data. To determine the particle size distribution and determine what proportion of various grains are present in a given raw powder sample, the standard sieving procedure outlined in ASTM D422/2014 was applied^15^. Following reporting, a semi-logarithmic graph is used to display the cumulative analysis. The volume-surface mean diameter (*Ds*) and median particle size (*D50*) are also determined. The powder density was measured using a standard water pycnometer (density flask) according to the standard ASTM D854/2024 method^16^.

##### Characterization of the CCMs

The physical properties of the CS membranes (cold and boiling water absorption, saturation coefficient, bulk density, and apparent porosity) were estimated according to ASTM C373/2024^17^, and the mechanical properties were determined for two different firing temperatures according to ASTM C109/2023^18^. The mechanical strength was evaluated using cubic specimens. Attenuated total reflectance Fourier transform infrared spectroscopy (ATR-FTIR) of the CCMs was performed for characterization of the membrane top layer using a Bruker VERTEX 80 (Germany) combined with platinum diamond ATR, which comprises a diamond disk as an internal reflector in the range of 4000–400 cm^−1^ with a resolution of 4 cm^−1^, refractive index of 2.4. This instrument is installed at NRC. The CCMs’ top surface and cross-sectional morphologies were examined by scanning electron microscopy (SEM) using a field emission gun (QUANTA FEG 250) instrument. Pore size distribution (PSD) of the CCMs was analyzed by a Pore Sizer (Micromeritics 9320, USA) installed at CMRDI. The surface charge of the composite membrane was measured using a zeta meter.

#### Membrane performance

##### Filtration test

The produced membranes’ permeability was evaluated at ambient temperature using pure water. The membranes’ pure water flux (PWF) was determined using Eq. (1)^19^.

1 $$PWF= \frac{V}{(A \times \Delta t)}$$

where V is the volume of filtrate (L), Δt is the operation time (h), and A is the effective membrane area (m^2^).

The laboratory-scale inorganic membrane filtration testing equipment employed in the current investigation, invented by CERAFILTEC in Germany, is a portable instrument designed for short-term filtering. It includes a membrane testing unit with a diameter of 5 cm, facilitating the testing of a relatively small sample. The wastewater used in the filtration experiment was used as a feed solution. Wastewater was gathered from the Kitchener drain in Kafr El-Sheikh Governorate, Egypt. The procedure involves submerging a single test plate in a 5 L feeding liquid reservoir containing agricultural effluent after pretreatment (screening) to remove larger particulates and debris before filtration. A pump is then used to draw contaminated water through the membrane unit for purification, with the purified permeate fluid collected in another tank. Backwashing must be performed between operating cycles with DI water and air to restore the membrane’s functionality and efficiency. Every experiment was run at a room temperature of 25 °C. The unit may replicate genuine filtration procedures such as filtration, backwash on-air, submerged backwash, and air scouring during or backwashing. The unit can operate continuously and automatically, with customizable settings, according to a control system built with Siemens LOGO and TDE software, which ensures precise and effective performance. The control system monitors variations in transmembrane pressure (TMP) with time, and the flow rate can be measured and customized via the control panel by regulating the pump speed.

The real samples of wastewater and treated wastewater were subjected to complete analysis at the National Water Research Center (NWRC). A PerkinElmer Optima 5300 DV Inductively Couple Plasma-Optical Emission Spectrometer (ICP-OES) was used to measure heavy metals before and after filtration using composite ceramic membranes (M2 and M4). The other parameters are estimated using standard methods^20^. The effectiveness of the CCMs in removing heavy metal ions was evaluated through experiments. Equation (1) was applied to measure the permeate flux. Equation (2) was used to calculate the heavy metal removal percentage.

2 $$R, \%=\left[\frac{\left({C}_{f}- {C}_{P}\right)}{{C}_{f}}\right] \times 100$$

where C_f_ is the feed concentration of heavy metals (mg L^−1^), C_p_ is the permeate concentration of heavy metals (mg L^−1^), and R is the examined removal (%)^21^.

##### Fouling test

A fouling experiment was performed using real wastewater. The primary membranes’ water flux (J_w1_) was measured using DI water for 60 min. Then, the permeate flux (J_P_) was calculated for 60 min. Every 15 min. of fouling variations in J_P_ were measured. After that, the contaminated membranes were cleaned using DI water for one h. The cleaned membranes’ water flux (J_w2_) was evaluated after stabilization for 60 min. The antifouling efficacy of the CCMs was assessed using the flux decline ratio (FDR) and flux recovery ratio (FRR), as indicated in Eqs. (3) and (4)^22^.

3 $$FDR= \frac{\left({J}_{W2}- {J}_{P}\right)}{{J}_{W2}}$$

4 $$FRR= \frac{{J}_{W2}}{{J}_{W1}}$$

## Findings and interpretations

### Characterization of the raw constituents

#### Elemental examination (XRF)

The components that are employed to create ceramic membranes have a significant impact on their properties. The chemical composition and loss of ignition (LOI) of the raw mix are identified in Table 1. Silica comprises most of the starting materials, followed by aluminum oxide. There are also small amounts of alkali and alkaline-earth metal oxides such as calcium oxide, magnesium oxide, potassium oxide, sodium oxide, and transition metal oxides (Fe_2_O_3_). The resulting composition is attributed to a combination of clays and quartz in the proposed raw mixture.

**Table 1:** Chemical analysis of the starting material.

Item	Wt. %
SiO_2_	62.7
Al_2_O_3_	26.2
Fe_2_O_3_	1.4
CaO	1.11
MgO	0.22
K_2_O	2.2
SO_3_	0.09
Na_2_O	0.37
L.O.I	5.71
Total	100.00

#### Mineralogical analysis (XRD)

**Fig. 2:**
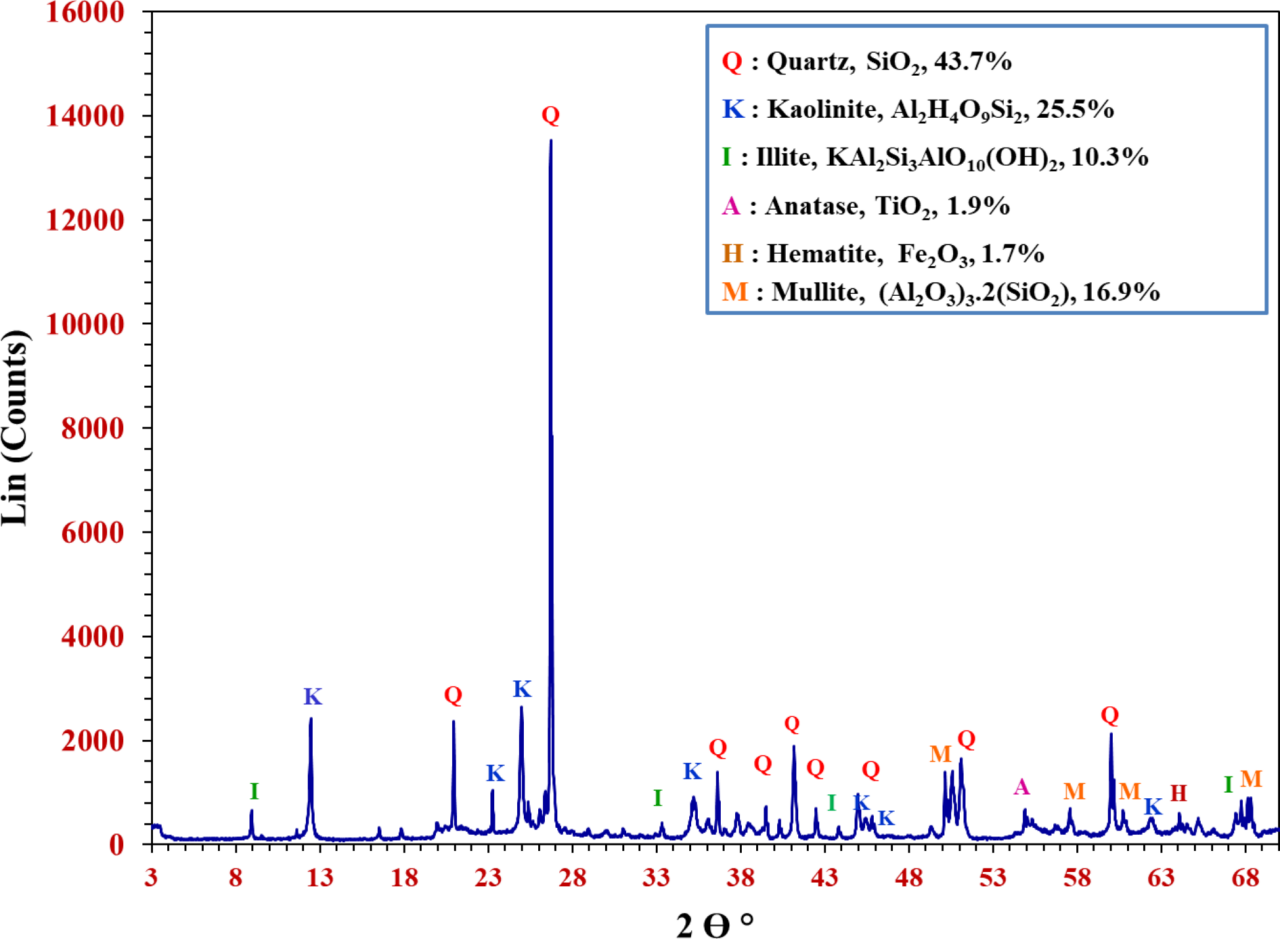
X ray diffraction pattern of the raw mix.

The XRD peaks show that the raw mix utilized is mostly made of quartz, SiO_2_ (ref. code: 00-005-0490), kaolinite, Al_2_Si_2_O_5_(OH)_4_ (ref. code: 9009230), and mullite, (Al_2_O_3_)_3_.2(SiO_2_) (ref. code: 00-070-0189), along with lesser quantities of illite, KAl_2_Si_3_AlO_10_(OH)_2_ (ref. code: 00-002-0056), anatase, TiO_2_ (ref. code: 01-071-1166), and hematite, Fe_2_O_3_ (ref. code: 01-089-0598). The observed peaks in the XRD patterns indicate that the mineral mixture formerly comprised quartz, kaolinite, and mullite as the major constituents, as shown in Fig. 2.

**Fig. 3:**
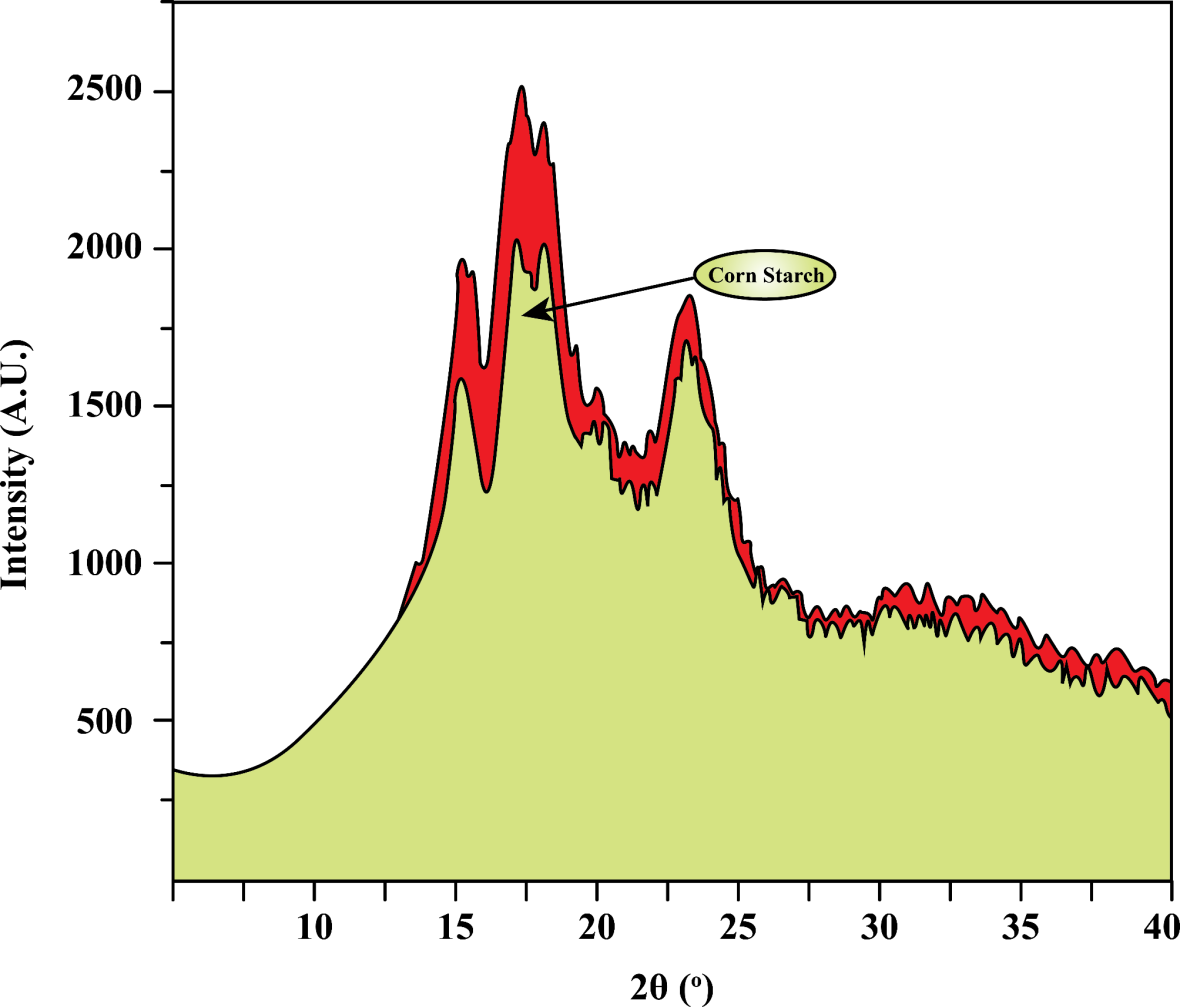
X ray diffraction pattern of corn starch^23^.

Figure 3 displays the XRD pattern of the corn starch used by Chen et al*.*^23^. The findings demonstrate that crystalline and amorphous components comprise the composition of corn starch. The crystallinity of the corn starch was 22.1%. The interactions within the interior starch molecule chains generate the crystal peaks. Through hydrogen bonding, the hydroxyl groups in the interior molecular chains of starch molecules assemble into chain crystals. A chain crystal created by a hydrogen linkage between the starch molecule chain and water is responsible for the peak near 22.8°^23^.

#### Thermal investigation

**Fig. 4:**
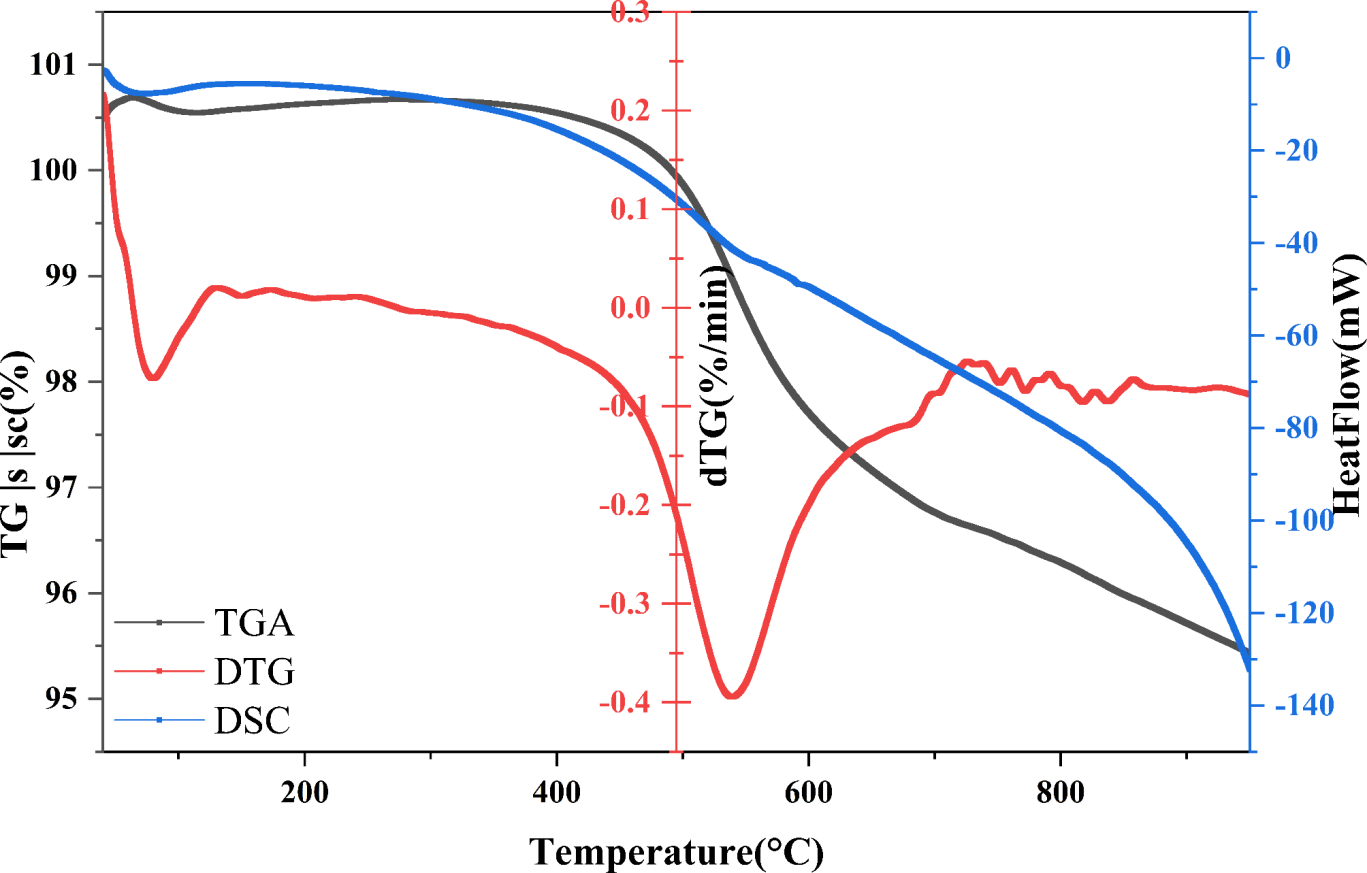
Thermal assessment curves for the starting mix.

The thermal behavior of the mixed powder was studied using TGA and DSC from an ambient temperature of 25 °C to 1000 °C, and the results are shown in Fig. 4. The weight loss percentages and the thermal stability of the primary material of the ceramic membrane were investigated using thermal analysis. Thermal analysis showed that the applied ceramic materials are stable at high temperatures. The DSC curve showed two main endothermic peaks. The earliest endothermic peak relates to the elimination of free and absorbed water. A 0.113 wt.% accompanies this dehydration process as a weight loss between 25 and 200 °C temperatures^24,25^. This peak can be associated with the predehydration procedure of kaolin. The reconfiguration of the octahedral layer, which starts at the surface’s OH, initiates the kaolin predehydration procedure^26^. The second DSC curve endothermic peak observed at approximately 580 °C resulted from the alpha–beta allotropic transformation of quartz^27^. The main weight loss of 5.216 weight percent that was detected between 400 and 580 °C is explained by alpha-quartz transforming into beta-quartz, and at 513 °C structural hydroxyl groups are lost when kaolinite transforms into metakaolinite, per the subsequent reaction^28^.

$${Al}_{2}{Si}_{2}{O}_{5}{\left(OH\right)}_{4} \left(450^\circ \text{C}-650^\circ \text{C}\right)= {\text{Al}}_{2}{\text{O}}_{3}.2{\text{SiO}}_{2} + 2{\text{H}}_{2}\text{O}$$

Throughout the thermal run between 25 °C and 1000 °C, the overall mass loss was 5.329%, consistent with the LOI of 5.71%.

**Fig. 5:**
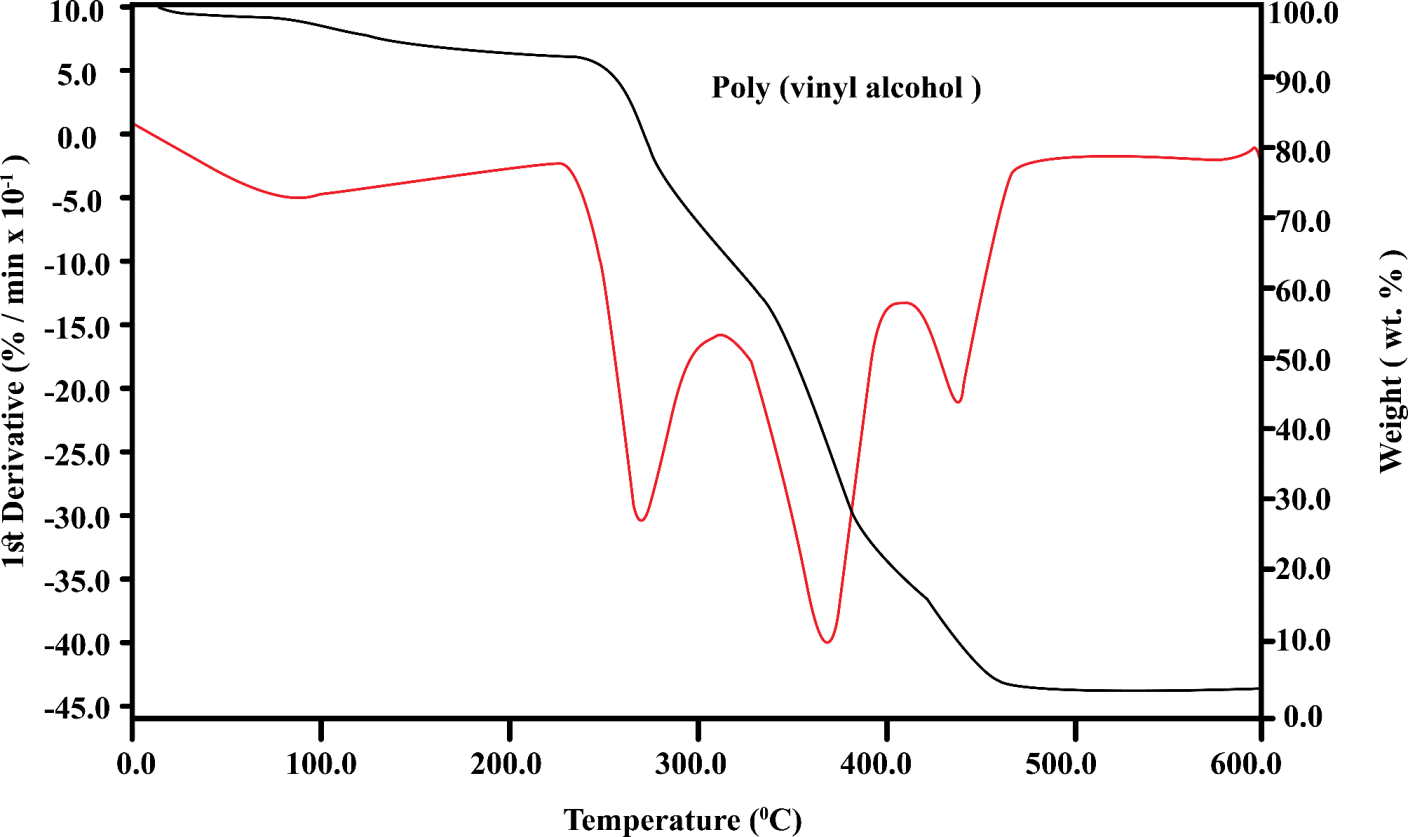
TGA of PVA^29^.

**Fig. 6:**
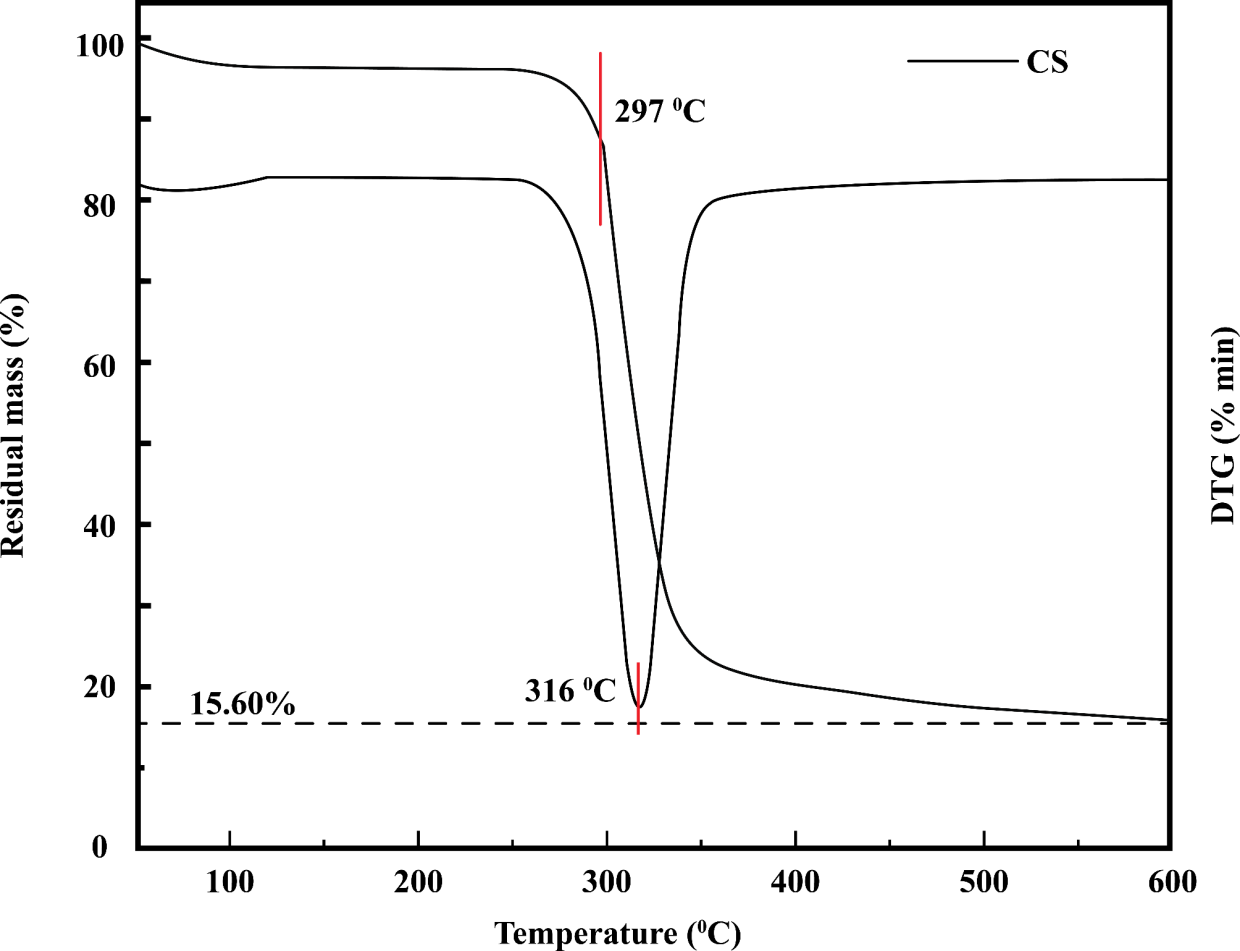
TGA of corn starch^23^.

The thermal behavior of PVA was studied by Gilman et al*.*^29^ and is displayed in Fig. 5. PVA is completely decomposed at approximately 480 °C^30^. Corn starch begins to break down at about 297 °C, as shown in Fig. 6. This is due to the breakdown of the α-1,4 glycosidic linkage, which initiates intermolecular dehydration. At 316 °C, corn starch is broken down at the most rapid rate. Corn starch completely broke down when the temperature approached 600 °C^23^.

#### The distribution of particle sizes

**Fig. 7:**
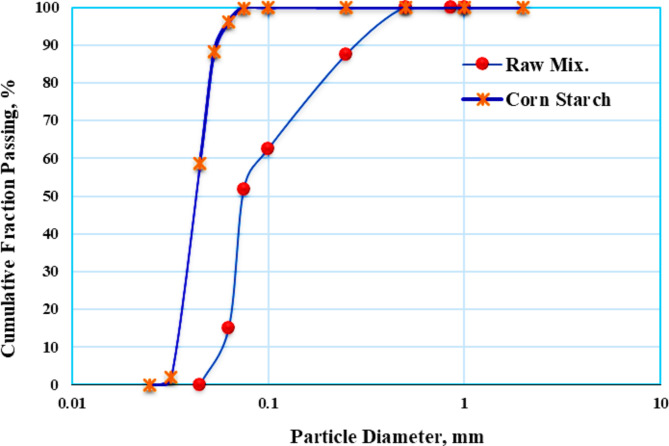
The cumulative curve for the grain size analysis of the used materials.

In ceramic membrane technology, the membrane’s pore size is highly dependent on the size of the initial material particles. Figure 7 presents the distribution of the grain size of the raw material. It illustrates that sixty percent of the raw mixed powder had particles smaller than 0.1 mm (100 µm).

The volume-surface average diameter *Ds*, or the measured diameter of the sphere with a similar volume/surface area proportion as a particle of concern, is used to calculate the fineness of the designed mixtures. The subsequent equation was utilized in its calculation^31^:

5 $$\overline{{D }_{S}}= \frac{1}{\sum_{i=1}^{N}\left(\frac{{x}_{i}}{\overline{{D }_{pi}}}\right)}$$

D_pi_ is the mean aperture of two successive screens, and xi is the mass proportion in a given increment.

The volume-surface average diameter (*D*_*s*_) was 0.09 mm (90 µm) for the raw mix and 0.086 mm (90 µm) for the corn starch. The median particle size (*D*_*50*_) was 0.075 mm (75 µm) for the raw mix and 0.043 mm (43 µm) for the corn starch.

#### The density of powder

The true density of the prepared mixture and corn starch was determined using a standard water pycnometer. The practices were repeated three times, and the average values were (2.22 ± 0.06) and (1.51 ± 0.03) g cm^−3^ for the raw mix and the corn starch, respectively.

### CCMs characterization

#### The mechanical and physical features

As shown in Table 2, the water absorption for M1 is greater than that for M3, which agrees with their apparent porosities; therefore, the porosity of M1 is greater than that of M3. The temperature of the sintering procedure greatly impacts porosity. When the temperature rises from 1000 to 1100 °C, the membrane porosity lowers from 40.78 to 39.70%. Partially densified ceramic material explains this phenomenon^32^. For the same explanation, as the temperature rises from 1000 to 1100 °C, boiling water absorption declines from 26.67 to 24.60, and cold water absorption lowers from 25.10 to 23.40. The membrane may have a significant permeability because of its high porosity. Densification takes place to create a durable ceramic body because, as this table demonstrates, the membrane density rises with the sintering temperature. Because silica is included in clay (46 weight percent), which partially melts during sintering, clay helps to densify ceramic structures at low temperatures^33^. The membrane particles are, therefore, tightly bonded to one another, indicating a high probability of an increase in mechanical strength as the sintering temperature rises. However, since bulk density and porosity have an inverse relationship with temperature, the bulk density results significantly verify the porosity findings.

**Table 2:** Mechanical and physical features of the constructed ceramic membranes.

Item	M1	M3
Sintering	T = 1000 °C Soaking time = 0.5 h	T = 1100 °C Soaking time = 3.0 h
Cold Water Absorption, %	25.10 ± 0.28	23.40 ± 0.21
Boiling Water Absorption, %	26.67 ± 0.61	24.60 ± 0.42
Saturation Coefficient	0.94 ± 0.02	0.95 ± 0.01
Bulk Density, g cm^−3^	1.53 ± 0.01	1.61 ± 0.02
Apparent Porosity, %	40.78 ± 0.41	39.70 ± 0.14
Compressive Strength, N mm^−2^	11.62 ± 0.57	15.44 ± 0.45

Also, Table 2 presents the mechanical strength of the prepared filters. As illustrated in the table, the strength of M3 is greater than that of M1 due to the higher sintering temperature, ranging from 1000 to 1100 °C. The amorphous silica phase’s densification and mullite’s crystallization are primarily responsible for this significant increase in mechanical strength^34,35^. The material is, hence, mechanically resistant. It is remarkable that these results fully align with the results of the XRD and SEM studies.

#### Surface functional groups

**Fig. 8:**
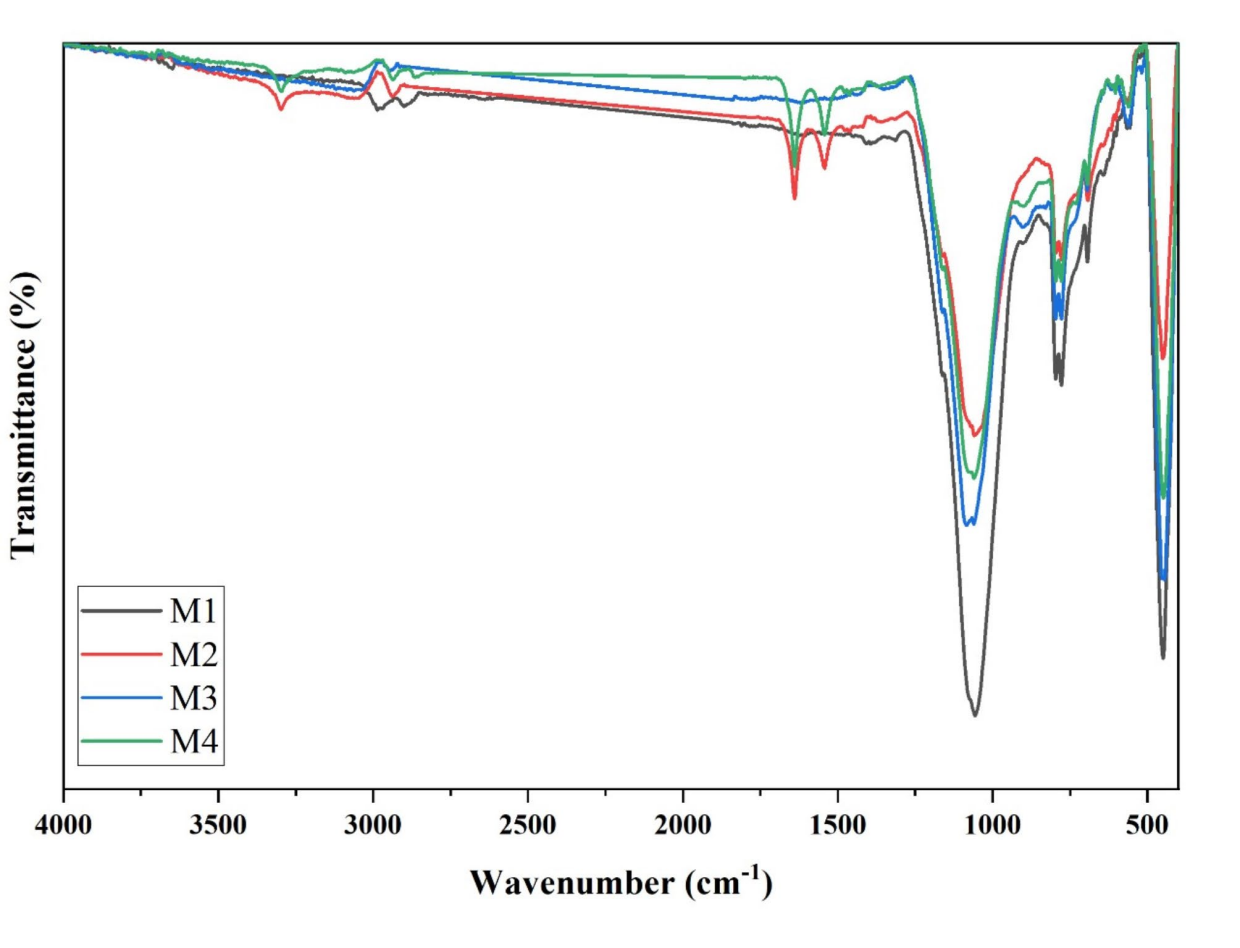
FTIR pattern of CCM without and with surface modification.

The FTIR approach can determine the top functional groups in support and modified ceramic membranes. According to the characterization obtained by ATR-FTIR (see Fig. 8), the polymer’s molecular band vibrations indicated that the polymer was present on the CS surface and that its chemical structure remained unchanged. The CSs were also subjected to FTIR analysis by an ATR system (Fig. 8) but did not exhibit polymer-region absorption bands, indicating that the PA6 spectrum is what was seen on the surface of the CCMs (Fig. 8).

Figure 8 shows the FTIR results of unmodified ceramic membranes; the band observed at 3062 cm^−1^ is assigned to the stretching of OH. H-O-H bending of water is detected at 1617 cm^−1^. The Si-O stretching of quartz is responsible for the peak noticed at 1084 cm^−1^^36^. OH deformation associated with Al^3+^ and K^+^ is attributed to the approximately 778–796 cm^−1^ peaks. It is attributed to the signal at 445 cm^−1^ to Si-O-Si asymmetrical bending^28^. The existence of bands at approximately 1084, 796, 778, 694, and 445 cm^−1^ revealed that each sample has a high quartz content. The peak observed at 1617 cm^−1^ is ascribed to the deformation vibrations of OH-adsorbed water. The peak observed at 903 cm^−1^ is assigned to Al-O–H vibrations; at 694 cm^−1^ is attributed to Si-O stretching and Si-O-Al stretching; and at 560 cm^−1^, Si-O bending and Si-O-Al stretching^37^.

As shown in Fig. 8, the FTIR results of modified ceramic membranes. The bands observed at 3707, 3296, and 2935 cm^−1^ are characteristic bands for the O–H band of formic acid, stretching of the hydrogen bond N–H of PA, and C-H axial deformation, respectively. The signals at 1639, 1542, 1463, and 1369 cm^−1^ correspond to the stretching of C═O, vibrations of N–H in-plane deformation, and vibrations of C-N stretching of amides, asymmetric deformation of C-H, and C-O stretching vibrations and carbon skeletons (amides), respectively. These bands are associated with PA6 and are placed upon the intrinsic bands of formic acid^38,39^.

#### SEM micrographs

**Fig. 9:**
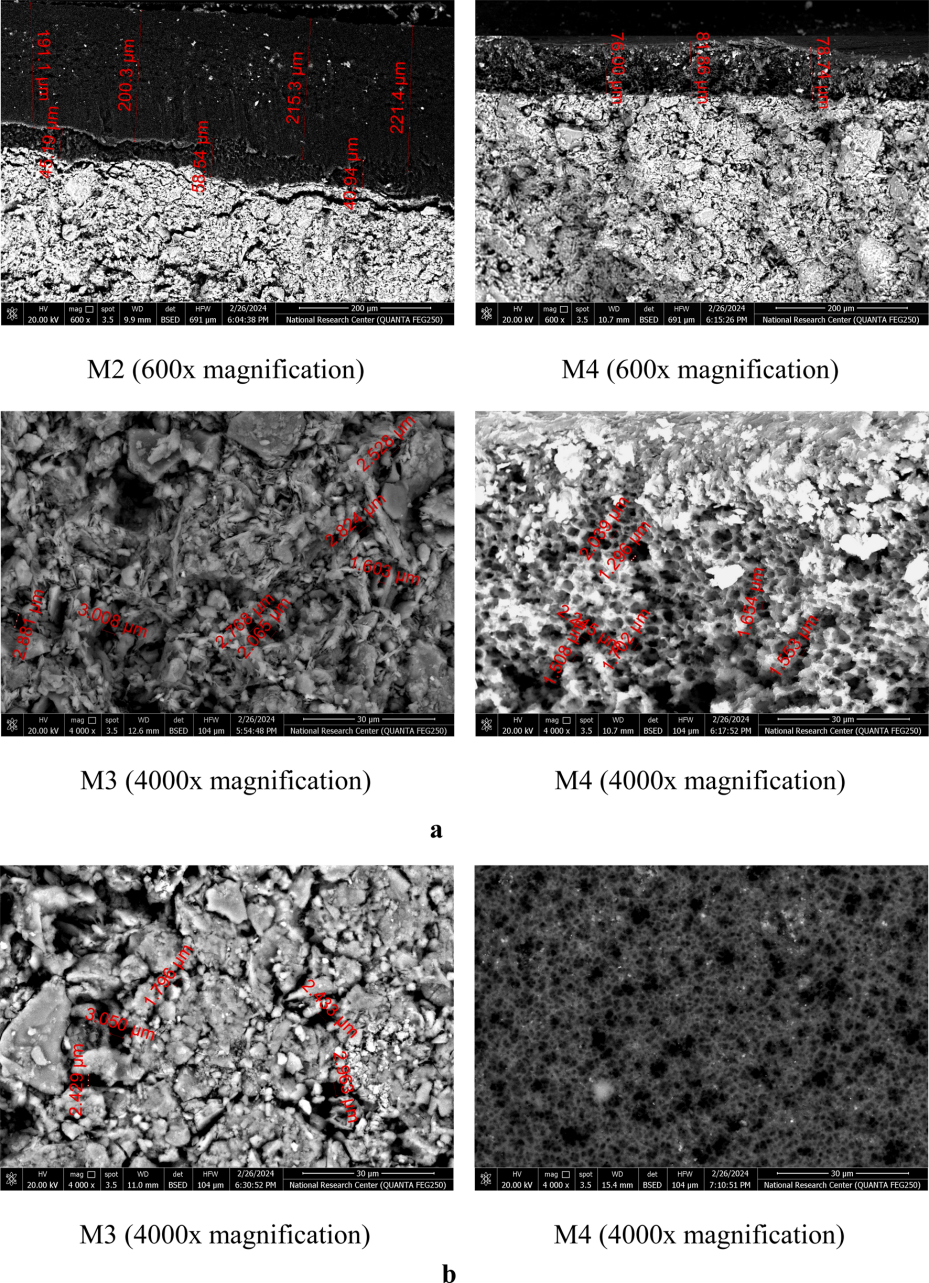
**a** Cross section micrographs of unmodified (M3) and Modified (M2 and M4) composite ceramic membranes. **b** Surface micrographs of composite ceramic membranes.

The results of the micrograph investigation (SEM) of the CMs and CCMs are presented in Fig. 9. SEM photographs of each membrane at different magnifications show that the membrane pore structure was random. The organic separation layers were successfully deposited onto the supports, as seen by the surface photographs of the CCMs. The penetration of the polymeric solution into the CS through an interfacial layer was demonstrated by verifying adequate coverage and filling of the pores in the membrane. The PA coating layer on the M2 support is denser (approximately 200 µm) than that covering the M4 support (approximately 80 µm). Generally, the deposited polymers cover the CS’s vacant areas. The results of the permeated flux further supported the polymer’s deposition and layer development on the CS^40^.

The adhesion between the microlayer and the support is excellent; therefore, there aren’t any cracks. This finding validates the optimal conditions while performing the slip-casting technique of the organic layer^27^. The morphology of the ceramic membrane is greatly influenced by the temperatures used for sintering, as seen by SEM photographs. At 1000 °C, sintering dominates and is sufficient to ensure satisfactory particle cohesiveness. This morphology provides a robust ceramic support. When the sintering temperature increased from 1000 to 1100 °C, the CS appeared homogenous with an adequate porous structure. The number of pores decreases owing to the fusing of tiny pores. Additionally, the partial vitrification of amorphous silica causes the onset of densification. This microstructure enhances the mechanical strength of the membrane but negatively affects the porosity^41^. As a result, the membrane has a denser shape and a notable degree of pore closure. The membrane sintered at 1100 °C is thought to have the maximum mechanical strength. These results explain why M2 and M4, modified with a thin layer of PA, had higher heavy metals separation efficiencies than unmodified membranes M1 and M3^35^.

#### The distribution of pore size

**Fig. 10:**
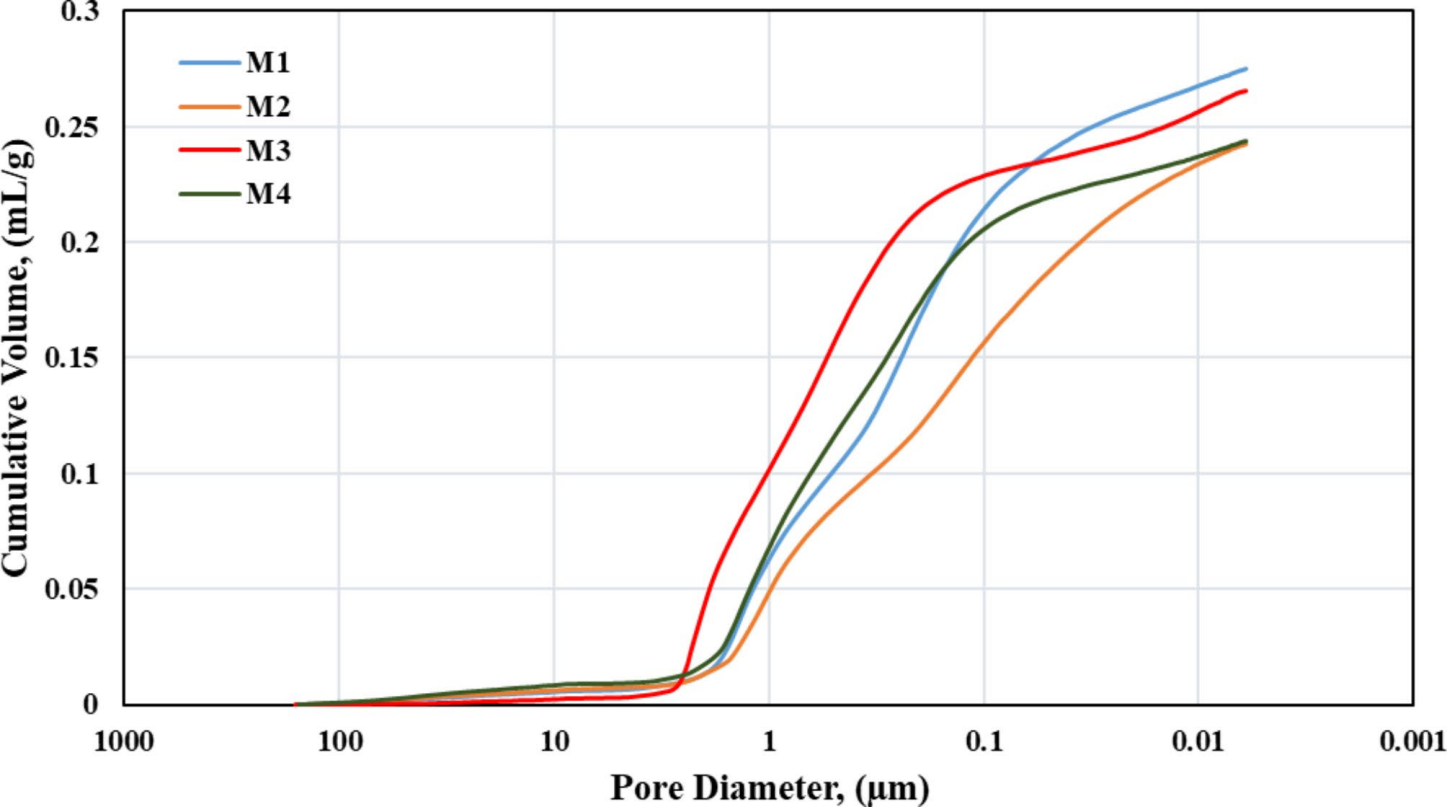
Cumulative pore volume for M1, M2, M3, and M4.

Mercury porosimetry was utilized to determine the pore size distribution (PSD), mean pore diameter, whole porosity, and entire pore area for produced membranes. The summary of the intrusion data is displayed in Table 3. The PSD is strongly associated with the sintering temperature. It decreases with increasing temperature, probably as the melting of minerals at higher temperatures may decrease the membrane’s pore size and total porosity^28,34^. Due to the large range, diverse membrane pore classes can be found along the PSD curve. Since all of the pores were assumed to be cylindrical objects, the average pore radius (r) is equal to 2V/A when the volume (V = πr^2^L) is divided by the pore area (A = 2πrL). The ratio of the total amount of mercury that has been intruded at the greatest pressure to the total volume of a sample is used to calculate porosity. The PSD for fabricated membranes are shown in Fig. 10. As shown in Table 3 and Fig. 10 the produced membranes have pore sizes ranging from 0.1 to 0.003 µm and average pore diameter from 0.09 to 0.05 µm, making them suitable for nano- and ultra-filtration applications. Pore diameters ranging from 0.005–0.01 µm are suitable for nano-filtration, whereas those between 0.01–0.1 µm can be used for ultra-filtration. This will allow it possible to eliminate smaller contaminants. Therefore, PA6 composite membranes may be an affordable economical replacement membrane.

**Table 3:** A summary of intrusion statistics.

Data	M1	M2	M3	M4
Total surface area of pores	13.138 sq-m g^−1^	16.454 sq-m g^−1^	11.315 sq-m g^−1^	9.890 sq-m g^−1^
Median diameter (volume) of pores	0.2783 µm	0.1942 µm	0.6577 µm	0.4494 µm
Median diameter (area) of pores	0.0183 µm	0.0176 µm	0.0115 µm	0.0144 µm
The average diameter of the pores	0.0985 µm	0.0838 µm	0.0939 µm	0.0589 µm
Bulk density	1.5776 g ml^−1^	1.5688 g ml^−1^	1.5988 g ml^−1^	1.5645 g ml^−1^
Apparent density	2.7875 g ml^−1^	2.5303 g ml^−1^	2.7784 g ml^−1^	2.5279 g ml^−1^
Porosity	43.41%	38.00%	42.46%	38.11%

### Efficiency of membranes

#### Separation experiments and wastewater treatment study

**Fig. 11:**
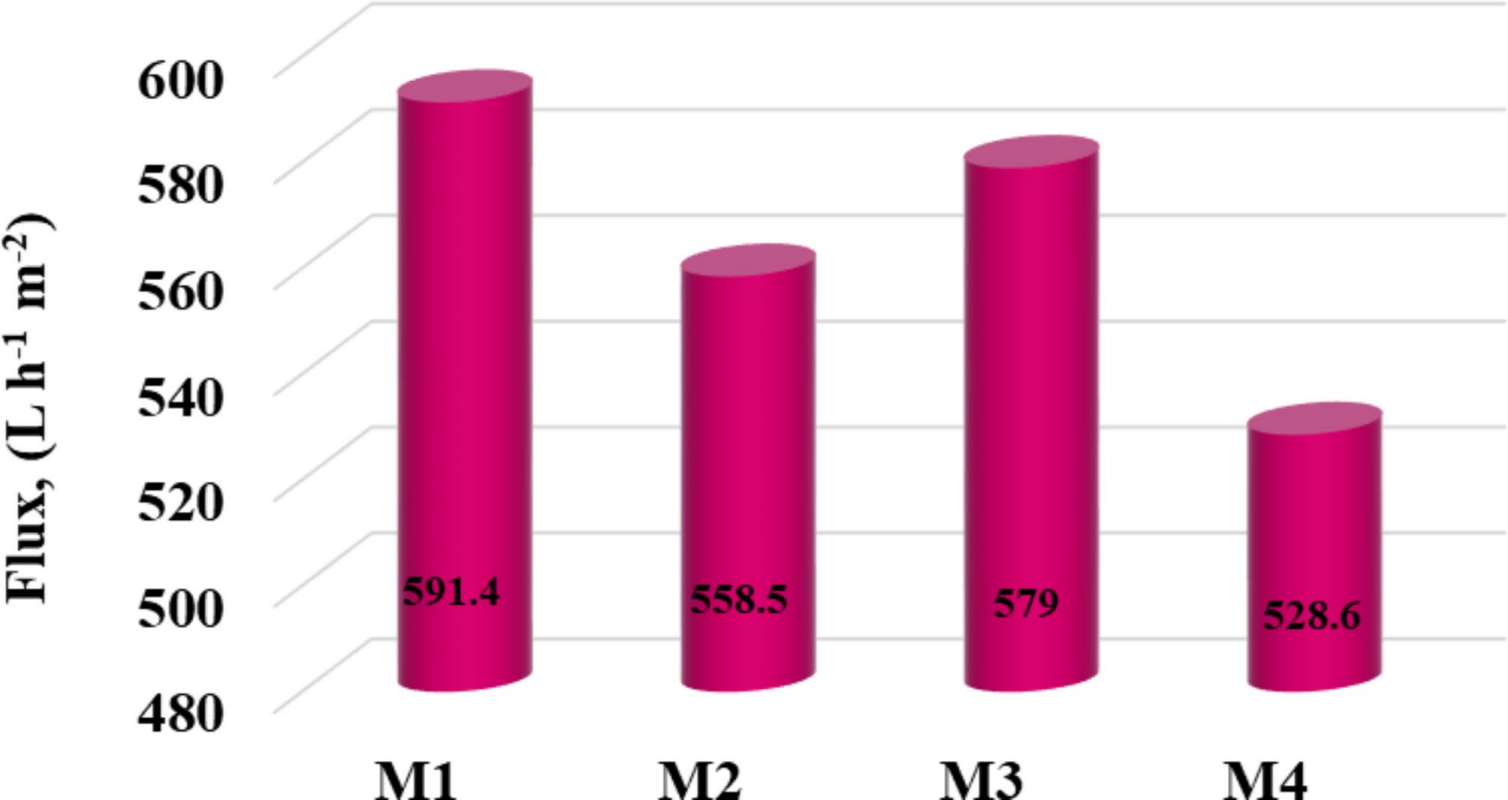
Water permeability of the produced membranes.

The water permeability Lp for the CSMs and CCMs is presented in Fig. 11. This diagram shows that the permeability decreases from 591.4 to 558.5 and from 579.0 to 528.6 L h^−1^ m^−2^ bar^−1^ at the same sintering temperature with the addition of a thin layer of polyamide. Given the significant correlation between permeability and porosity, the decline in permeability values might be attributed to a decrease in porosity following polymer addition^42^. Additionally, the permeability decreases due to a decrease in membrane porosity with increasing sintering temperatures. Good adhesion between the separating layer and the CS is essential for the structural stability of the CCMs, according to Wei et al*.*^43^. The composite membrane remained stable throughout the testing, per the permeability findings previously reported.

As predicted, the CS showed a greater water flux. Pure water flux decreased as a result of the top layer of PA6 in the composite membrane, demonstrating how polymer deposition on the CS’s surface enhanced the barrier to water flux^44^.

**Fig. 12:**
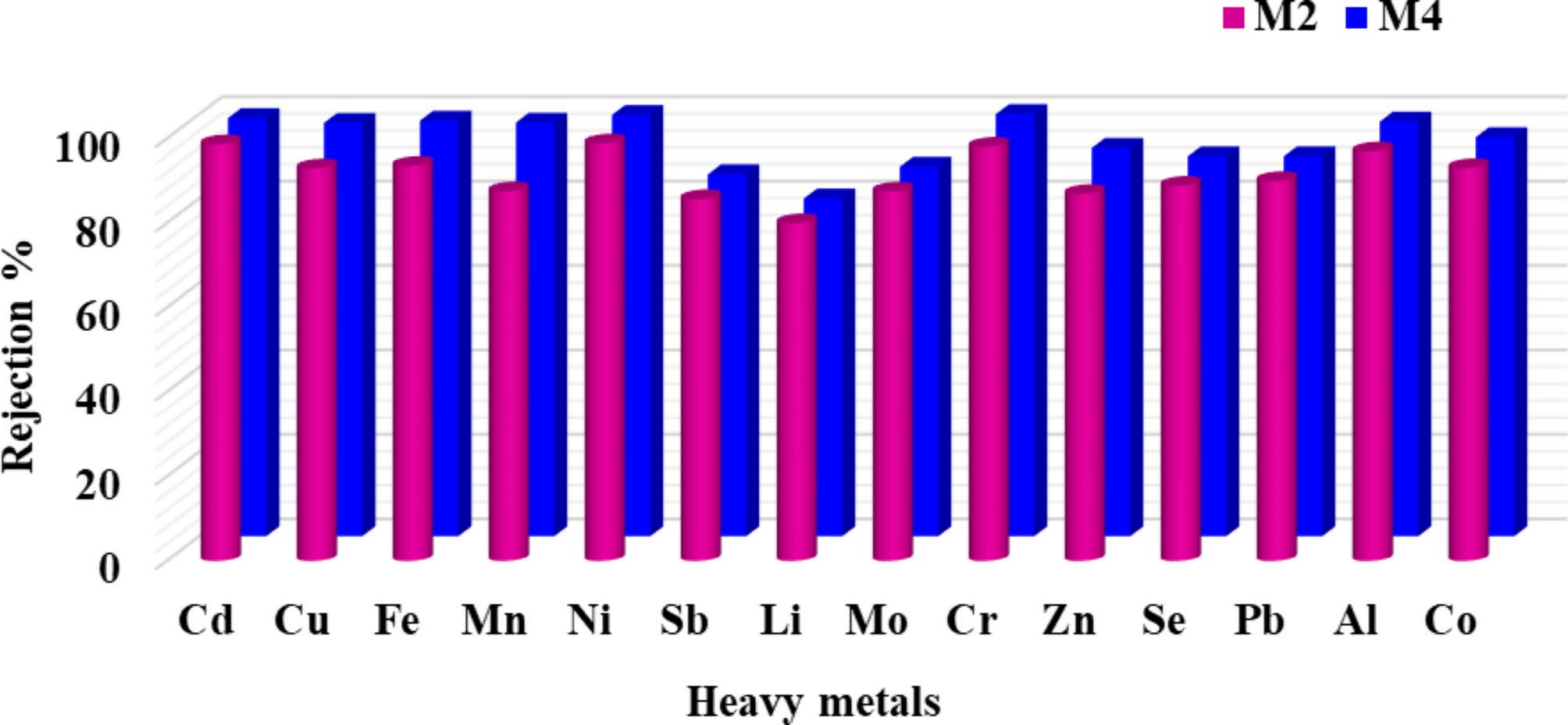
Separation efficiency of modified ceramic membranes for removing heavy metals from wastewater.

A rejection study of heavy metals and other contaminants present in agricultural wastewater was performed using two modified ceramic membranes (M2 and M4). With an increase in sintering temperature, the permeate flux decreased. The permeate fluxes were 341.07 and 276.35 L h^−1^ m^−2^ for M2 and M4, respectively. CCMs exhibited greater efficiency in removing heavy metals from Kitchener wastewater. Table 4 displayed the characteristics of both the wastewater and the permeate following the filtration experiments via M2 and M4; the removal efficiencies reached 97%, as shown in Fig. 12. The interaction of the effluent with the membrane contributes to the greater retention rates of heavy metals^45^. The CCMs have a pH_zpc_ value of 5.8. According to this finding, the membrane is positively charged at pH values below 5.8 and negatively charged at pH values over 5.8. Steric hindrance can occur when certain metal ions chelate with the PA membrane’s surface amine groups, preventing other metal ions from passing through even at high pressure^46^. The rate at which metal ions are removed is accelerated when positively charged heavy metals adsorb on the negatively charged membrane surface. Metal cations tend to chelate with the -NH_2_ groups on the membranes due to deprotonated amine groups, reducing the positive charge on the membrane surface. Thus, the Donnan effect may also explain why heavy metal cations are rejected faster^47^.

**Table 4:** Analysis of real wastewater before and after treatment with composite ceramic membranes.

Parameter to be analyzed	Units	Wastewater analysis before treatment (Feed analysis)	Water analysis after treatment using M2	Water analysis after treatment using M4	Accepted limits (according to the decree of the Ministry of Health 458/2007)
**[1] Physical parameters**					
Colour	-	Yellowish	colorless	colorless	colorless
Turbidity	N.T.U	3.7	< 0.01	< 0.01	1
Odour	-	Fish Odour	Odorless	Odorless	Odorless
**[2] Physicochemical parameters**					
pH	-	7.4	7.2	7.1	6.5 – 8.5
Conductivity	µS/cm	5007	3603	3590	-
Total hardness as (CaCO_3_)	mg/l	1225	17	11	500
Calcium (Ca^++^)	mg/l	232	2.66	2.07	350
Magnesium (Mg^++^)	mg/l	162	3.28	2.48	150
Bicarbonate (HCO_3_^−^)	mg/l	273	7	4	-
Sodium [Na^+^]	mg/l	858	618.8	497.6	200
Potassium [K^+^]	mg/l	36	24.7	20.8	-
Chloride [Cl^−^]	mg/l	1421	108	94.7	250
Sulphate [SO^4−^]	mg/l	875	9	8	250
Total dissolved solids [TDS]	mg/l	3210	1873	1856	1000
Biological oxygen demand [BOD]	mg/l	32	< 0.01	< 0.01	-
Chemical oxygen demand [COD]	mg/l	41	< 5	< 2	-
Total organic carbon [TOC]	mg/l	39	Not detected	Not detected	-
Total volatile suspended solids [TVSS]	mg/l	32	< 0.01	< 0.01	-
**[3] Undesirable substances**					
Ammonium [NH^4+^]	mg/l	2.2	< 0.001	< 0.001	0.5
Nitrates [NO_3_^−^]	mg/l	15.8	0.4	0.1	45
Nitrite [N]	mg/l	0.38	< 0.001	< 0.001	0.2
Phosphate [PO_4_]	mg/l	6.5	< 0.001	< 0.001	-
Silica [SiO_2_]	mg/l	15.8	< 0.001	< 0.001	-
Iron [Fe]	mg/l	3.120	0.200	0.050	0.3
Manganese [Mn^++^]	mg/l	11.250	1.400	0.230	0.4
Copper [Cu^++^]	mg/l	2.850	0.200	0.060	2
Zinc [Zn^++^]	mg/l	0.375	0.049	0.030	3
Aluminum [Al]	mg/l	3.350	0.100	0.060	0.2
Lead [Pb]	mg/l	0.010	0.001	0.001	0.01
Cadmium [Cd]	mg/l	45.750	0.590	0.450	0.003
Nickel [Ni]	mg/l	8.250	0.090	0.020	0.02
Cobalt [Co]	mg/l	102.750	7.130	5.630	-
Selenium [Se]	mg/l	0.450	0.050	0.045	0.01
Chromium [Cr]	mg/l	3.210	0.060	0.001	0.05
Molybdenum [Mo]	mg/l	0.080	0.010	0.010	0.07
Lithium [Li]	mg/l	0.050	0.010	0.010	-
Antimony [Sb]	mg/l	0.070	0.010	0.010	0.02

#### Composite membrane fouling

**Fig. 13:**
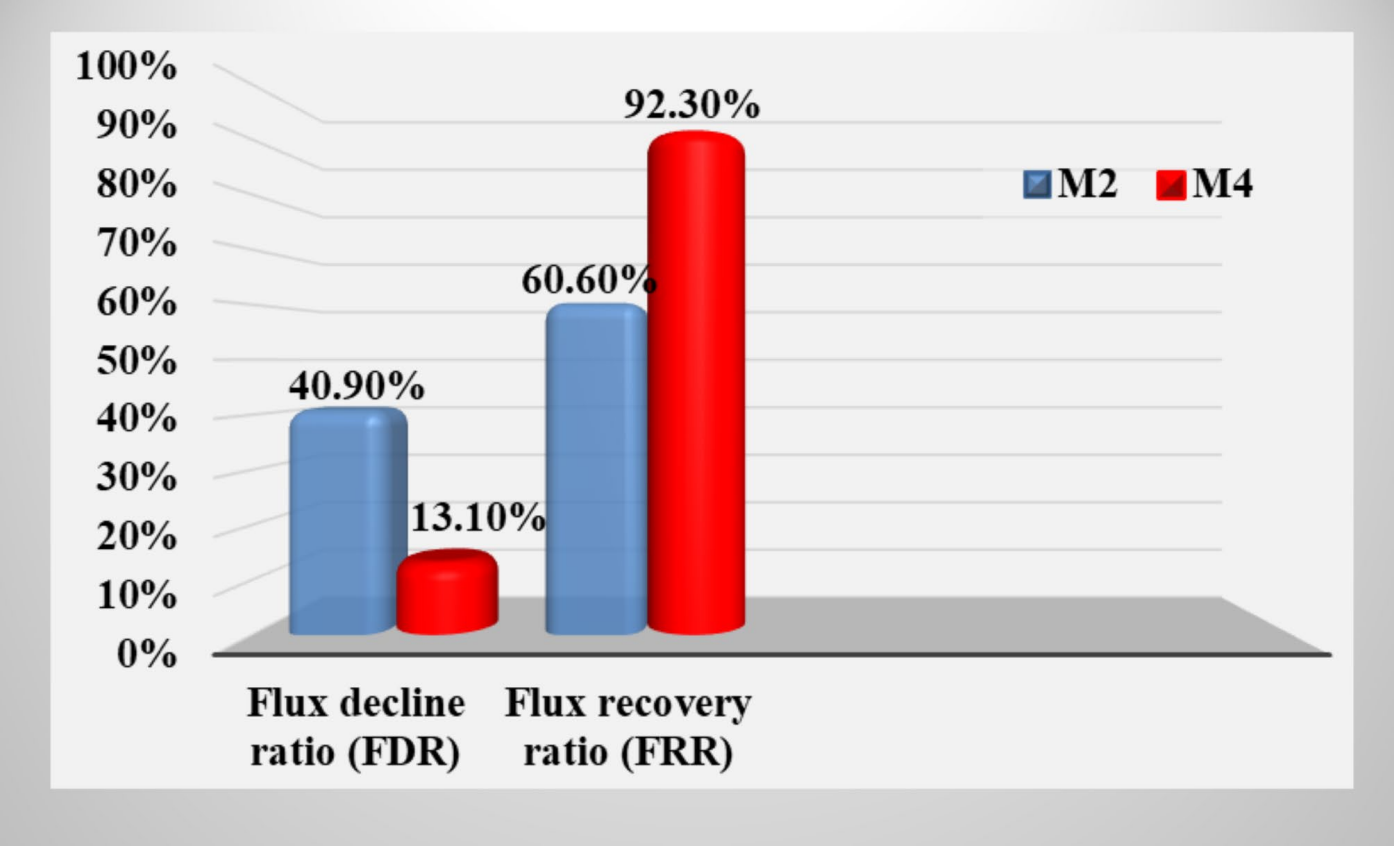
Flux decline and flux recovery ratios of fouling tests for modified membranes M2 and M4.

**Fig. 14:**
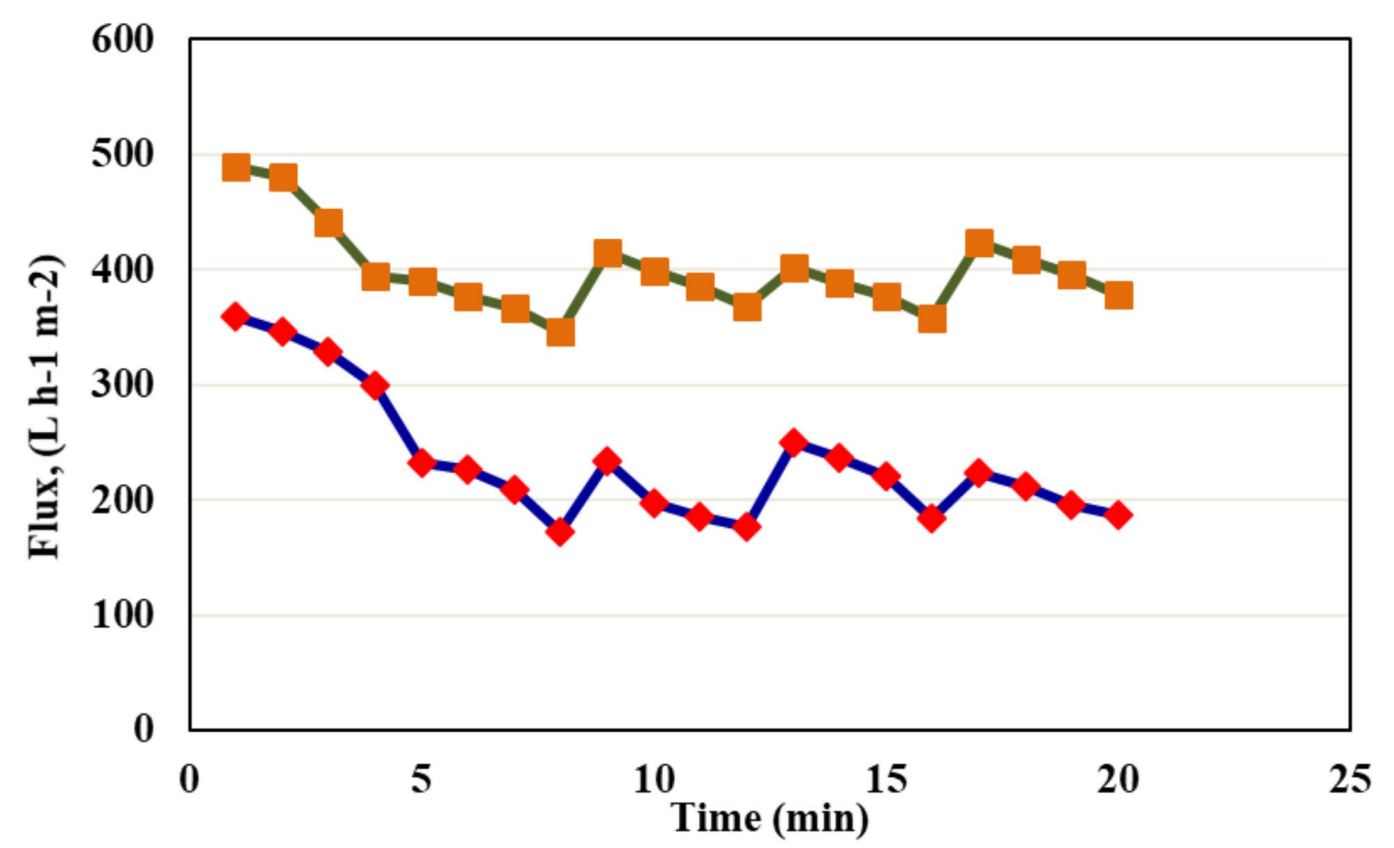
Antifouling test for M2 and M4.

Fouling in separation procedures is generated primarily by heavy metal deposition on the barrier’s surface and through the pores within the membrane. For this test, water was used to wash each membrane for 15 min. The FRR and FDR ratios for M2 and M4 are shown in Fig. 13. As demonstrated in Fig. 14, cleansing with only clean water was sufficient to eliminate most heavy metals adsorbed on the membrane’s pores and surface^48^.

#### The economic aspect of produced membrane

It is critical to evaluate the cost of any process to determine its practical feasibility. Ceramic membranes are typically priced between $500 and $3000 per square meter^49^. In this study, we assessed the cost of raw materials and energy utilization for manufacturing ceramic membranes (both unmodified and modified). Employing inexpensive resources that do not require high sintering temperatures can reduce the cost of ceramic membranes, making them more cheap. However, it is vital to highlight that any novel and low-cost ceramic membrane must be reliable, stable, and robust. Based on the raw materials expenses (Table 5) and the consumption of energy (Table 6) used to create the ceramic membranes in this investigation, membranes M1, M2, M3, and M4 were estimated to cost approximately 12, 119, 16, and 122 USD/m^2^, respectively. These estimations imply the possibility of constructing ceramic-based membranes at considerably lower prices, making them more economical and accessible for a wide range of wastewater treatment applications. The cost of ceramic membranes considerably impacts their widespread use, particularly in large-scale applications. By lowering production costs, a wider range of industries and applications can benefit from ceramic membranes’ distinctive characteristics, such as high selectivity, durability, and tolerance for harsh conditions.

**Table 5:** Cost analysis study of raw materials for membranes in USD.

Raw materials	Price (USD per kg)	The raw material used (g) per m^2^ of membrane	Cost of raw material per m^2^ of membrane (USD)	Cost per m^2^ of membrane (USD)	
				M1 or M3 (ceramic membrane)	M2 or M4 (modified membrane)
Ball clay	0.1	55	0.0055	0.16511	106.73311
Potash feldspar	0.4	44	0.0176		
Quartz sand	1.00	19.8	0.0198		
Kaolin	0.50	46.2	0.0231		
Corn starch	0.01	11	0.00011		
PVA	15	6.6	0.099		
PA6	476	220	104.72		
FA	2 USD per liter	869	1.738		
EDA	10 USD per liter	11	0.11

**Table 6:** Fee for electricity utilization.

	Device	Consumption time (h)	Energy utilization (kWh per hour)	Energy utilization to prepare m^2^ of membrane (kWh)	Cost based on average global electricity price (USD)
Laboratory ball mill		0.5	1.25	0.625	0.078125
Laboratory hydraulic press		0.25	1.5	0.375	0.046875
Laboratory drying oven		12	7.3	87.6	10.95
Magnetic stirrer		0.5	0.35	0.175	0.021875
Muffle furnace	M1 or M2	0.5	11	5.5	0.6875
	M3 or M4	3		33	4.125
Total	M1 or M2			94.275	11.784375
	M3 or M4			121.775	15.221875

## Comparative study

As shown in Table 7, this enhanced table compares different composite membranes for heavy metal removal from aqueous solutions, including their performance in heavy metal rejection, water permeance, and fouling resistance.

**Table 7:** Comparison of heavy metals removal composite membranes with previously reported study.

Membrane materials	Feed concentration	Target metals	Separation mechanism	Water flux (L m^−2^ h^−1^)	Removal efficiency	Fouling resistance/flux recovery ratio	References
Graphene oxide/onion extract composite	1.0 M	Cr(VI) , As(III), Cd(II), and Pb(II)	Surface Charge repulsion	460	(88–100) %	Not specified	^50^
Graphene oxide/quercetin composite	1.0 M	Cr(VI) , As(III), Cd(II), and Pb(II)	Surface Charge repulsion	150	(78–95) %	Not specified	^50^
Poly vinylidene fluoride/ionic liquid reduced graphene oxide nanocomposite	10 ppm	Cu(II), Cd(II), Zn(II), Mn(II), and As(III)	Surface Charge repulsion	412.2	(70–90) %	94%	^51^
microbial amyloid nanofibers	50 ppm	Pb(II)	Adsorption	-	98%	Not specified	^52^
Mxene/Carbon composite-based nanofibers	10 ppm	Pb(II), and As(III)	Adsorption	-	(81–89) %	Not specified	^53^
Amino-functionalized graphene oxide nano-sheets/polyethersulfone	Not specified	Cu(II), Pb(II), and Cd(II)	The Donnan ion effect	46.57	(92–95) %	72.46%	^54^
PA6 /ceramic composite membrane	(2.85–102.75) ppm	Cu(II), Fe(III), Mn(II), Cd(II), Ni(II), Cr(VI), Al(III), and Co(II)	The Donnan effect and chelation with surface amino group	528.6	(80–100) %	92.3%	This study

It would be acceptable to state that this CCM displayed high separation efficiency for eliminating heavy metals from real wastewater with a high flux recovery ratio and pure water flux compared to the latterly described composite membranes (Table 7). This could be explained by some characteristics of the modified ceramic membrane, such as charged surface active layer, porous structure, mechanical properties, and permeability.

## Conclusions

Treatment of agricultural wastewater is mandatory because it contains a variety of heavy metals; these heavy metals are very toxic to human and aquatic life. In addition, these elements, even at low concentrations, are harmful to plants, where they can be released into the soil. Also, heavy metals at minimum concentrations destroy microorganisms found in municipal wastewater treatment plants. So, highly efficient CCMs have been effectively fabricated from locally accessible raw materials with negligible commercial value. The membranes were modified with a thin film of PA6 via the dip coating method. The newly prepared membranes showed very good physical and mechanical properties. The CCM formed, M4, achieved highly efficient performance in removing many heavy metals in real wastewater from the Kitchener drain, reducing up to 99.97%. The membrane, M4, showed a better flux recovery ratio and a very low flux decline ratio of 92.3% and 13.1%, respectively. The produced CCMs are cheap, easy to prepare, and easy to reuse, where fouling is limited (eco-friendly membrane). This modified membrane has proved to be efficient in the treatment of agricultural wastewater (i.e., it contains a variety of contaminants at different concentrations). This modified membrane can be recommended as an efficient one for the treatment of agricultural wastewater; the membrane fouling is limited; it can be reused, whether in agriculture or some industrial applications, which leads to the preservation of natural resources. Thus, it helps to achieve sustainable development goals.

## Data Availability

All the data generated or analyzed during this study are included in this published article.
